# Mining FDA drug labels for medical conditions

**DOI:** 10.1186/1472-6947-13-53

**Published:** 2013-04-24

**Authors:** Qi Li, Louise Deleger, Todd Lingren, Haijun Zhai, Megan Kaiser, Laura Stoutenborough, Anil G Jegga, Kevin Bretonnel Cohen, Imre Solti

**Affiliations:** 1 Division of Biomedical Informatics, Department of Pediatrics, University of Cincinnati, Cincinnati, OH, USA; 2 Computational Bioscience Program, University of Colorado School of Medicine, Aurora, CO, USA; 3 Anderson Center for Health Systems Excellence, Cincinnati Children's Hospital Medical Center, Cincinnati, OH, USA

**Keywords:** Medical condition, Disease and disorders, Sign and symptoms, cTAKES, NLP, Natural language processing, IE, Information extraction, CRF, Conditional random fields, FDA drug labels

## Abstract

**Background:**

Cincinnati Children’s Hospital Medical Center (CCHMC) has built the initial Natural Language Processing (NLP) component to extract medications with their corresponding medical conditions (Indications, Contraindications, Overdosage, and Adverse Reactions) as triples of medication-related information ([(1) drug name]-[(2) medical condition]-[(3) LOINC section header]) for an intelligent database system, in order to improve patient safety and the quality of health care. The Food and Drug Administration’s (FDA) drug labels are used to demonstrate the feasibility of building the triples as an intelligent database system task.

**Methods:**

This paper discusses a hybrid NLP system, called AutoMCExtractor, to collect medical conditions (including disease/disorder and sign/symptom) from drug labels published by the FDA. Altogether, 6,611 medical conditions in a manually-annotated gold standard were used for the system evaluation. The pre-processing step extracted the plain text from XML file and detected eight related LOINC sections (e.g. Adverse Reactions, Warnings and Precautions) for medical condition extraction. Conditional Random Fields (CRF) classifiers, trained on token, linguistic, and semantic features, were then used for medical condition extraction. Lastly, dictionary-based post-processing corrected boundary-detection errors of the CRF step. We evaluated the AutoMCExtractor on manually-annotated FDA drug labels and report the results on both token and span levels.

**Results:**

Precision, recall, and F-measure were 0.90, 0.81, and 0.85, respectively, for the span level exact match; for the token-level evaluation, precision, recall, and F-measure were 0.92, 0.73, and 0.82, respectively.

**Conclusions:**

The results demonstrate that (1) medical conditions can be extracted from FDA drug labels with high performance; and (2) it is feasible to develop a framework for an intelligent database system.

## Background

Every year, more than 2 million patients suffer serious Adverse Drug Reactions (ADRs) in the US; of those, 100,000 reactions are fatal [1]. Due to this fact, the majority of previous work on detecting healthcare-related adverse events has focused on identifying ADRs. ADRs have been ranked among the top four causes of death in the US [2]. Cincinnati Children’s Hospital Medical Center (CCHMC) has over 1 million patient-physician encounters a year. To scale ADR detection to an encounter-volume this high, CCHMC is engaged in a larger patient safety research project and has implemented a trigger-tool based approach for a selected set of drugs [3]. In the case of trigger-tools, an algorithm monitors the electronic patient records for a drug-specific trigger (e.g. administration of Naloxone to counter opiate overdose) and alerts hospital management for potential ADRs. We describe a use-case for developing an intelligent ADR database system by extracting medications and their corresponding medical conditions from FDA drug labels. It would be optimal to extend the automated trigger-tool methodology to an all-inclusive set of drugs; however, current trigger-tool methodology requires manual database development for the triggers. In other words, a human user needs to enter ADR signs and symptoms into a database that serves as the underlying data set to scan the EHR content for ADR triggers.

We define a database system to be intelligent if it can be self-sustaining and stay current with little or no human intervention. Our aim in this paper is to present the results of building the first NLP-based component of a proposed intelligent database system. This intelligent database system could gather information from multiple publicly-available sources, while continuously updating itself after the initial setup. FDA drug labels could serve as one of the main sources for candidate ADR data. Additionally, Adverse Event Reporting System (AERS) [4], full-text journal articles, and data with statistical associations between drugs and signs and symptoms collected from the EHR itself can also be resources. The FDA publishes drug labels for most over-the-counter (OTC) and prescription (Rx) drugs (37,907 drugs altogether, as of 11/05/2012). As the FDA states on its web site, “*FDA-approved drug labels contain a wealth of information about ADRs from clinical trials and post-marketing surveillance. The information included in the labels is agreed upon by regulatory, industry, and consulting experts who have incorporated their contributions over decades, reflecting the best thinking at the time. Thus, the drug labelling implicitly balances the information of causality, incidence, and severity based on 1) data from controlled trials, 2) published literature reports, and 3) spontaneous reports to AERS (adverse event reporting systems)*” [5].

Although as Boyce et al. have pointed out [6], drug labels are not a perfect source of medication-related medical information, there is nonetheless a wealth of information in the labels that represents important knowledge with strong implications for patient safety and the quality of health care. Chen et al. applied FDA-drug labels for detecting drugs that can induce liver injury [7]. Murphy et al. also stated, *“The information included in the labelling is the ‘true scoop’ and… the closest one can get to the truth regarding the scientific information known about a drug. The information contained in the package insert or product information is the result of careful and extensive analyses performed by the company and then the FDA, using source data”*[8]. In this paper, as a use case for building an intelligent database, we present our approach to “unearthing” the information buried in the FDA label’s narrative text using natural language processing (NLP) methodology. We also test two hypotheses: that medical conditions can be extracted from FDA drug labels using scalable NLP methods, and that the open source clinical Text Analysis and Knowledge Extraction System (cTAKES) can be used for textual genres other than clinical texts.

At the current time, the most common method of ADR surveillance, within most healthcare organizations, is largely a manual effort. Manual chart review techniques require experts (e.g. physicians and nurses) to read the clinical notes of patients to parse out pertinent safety information. This is time-consuming [9]. Moreover, as such chart review methods do not scale well to large bodies of data, institutions usually utilize sampling methods (i.e., randomly choosing portions of records) for ADR detection. Phansalkar et al. found that pharmacists detected higher rates of adverse drug events than non-pharmacists [10]. The chart review approach not only requires the experts to have comprehensive knowledge of the administered medications and their associated indications, contraindications, and ADRs, but also demands that the human reviewer be intimately familiar with all details of the patients in order to be able to judge if a new diagnosis, sign, or symptom recorded in the notes is a potential ADR signal. The FDA previously developed the Coding Symbols for Thesaurus of Adverse Reaction Terms (COSTART) [11], which was used for coding, filing, and retrieving post-marketing adverse drugs and biologic experience reports. COSTART was then superseded by the Medical Dictionary for Regulatory Activities (MedDRA) Terminology [12]. MedDRA is endorsed by international organizations and is used in multiple countries for safety reporting from pre-marketing to post-marketing activities, as well as medication-related data entry, retrieval, evaluation, and presentation. These two systems are usually used for medication coding, but do not provide methods for automatically extracting medical conditions.

Some subsequent work used these dictionaries for further medical condition extraction. He et al. proposed the Adverse Event Ontology (AEO) to represent adverse events [13]. However, AEO only covers 484 representation units, whereas MedDRA comprises 70,177 lowest-level terms in version 15.0. In addition to MedDRA, SNOMED CT was also successfully used to code terms as potential ADRs. Alecu et al. demonstrated that SNOMED CT codes, with cross-mapping by UMLS, can be successfully translated into the MedDRA schema. SNOMED CT has the advantage that it is fine-grained, with detailed definitions of terms [14], so it is included in our FDA label extraction results. The ADESSA system, a real-time decision support service for detecting or monitoring adverse drug events, uses a regular expression and dictionary-based method to extract ADRs [15,16]. This system shows the viability of mining FDA labels to discover ADR terms; it relies on MedDRA as a dictionary to extract terms that represent ADRs in the drug labels. Although the developers of ADESSA reported good performance, the details of the regular-expression algorithm and the evaluation are not provided. Moreover, even if the regular-expression-based method has been found to be useful for a variety of tasks, the limitation of this method is that if the ADR terms are not recorded in the dictionary, then the algorithm is not likely to detect them. In addition, term variability will limit the usefulness of this method. Although cTAKES has an ADR detection module [17], we did not use it because the module was custom developed for the text of clinical notes and not FDA labels, whose texts are quite different. Moreover, we expected to collect indications, contraindications and overdosage, as well, which are not extracted as ADRs by the cTAKES system.

A machine learning algorithm with contextual features has a chance to discover terms missed by dictionary-based methods. Bisgin et al. successfully implemented an unsupervised machine learning technique, Latent Dirichlet Allocation (LDA), for topic modelling on FDA labels [18]. They demonstrated how topic modelling via unsupervised techniques could contribute to drug safety. However, this kind of unsupervised learning method can only detect the topics, not the entities. Semi-supervised learning methods have been applied in named entity detection. For example, Li et al. used a bootstrapping method to detect named entities of locations and products in an industry corpus [19]. Although this method can achieve high precision, recall is low, especially when the size of sample seeds is small. Supervised learning methods reported the highest overall performance. Aramaki et al. used a supervised learning method to extract adverse drug events from Japanese clinical records. Extracting drugs and symptoms from Japanese clinical notes is quite different from disease/disorders and sign/symptoms extraction from English drug labels, especially concerning the features for supervised learning [20]. We decided on a supervised method to serve as the core of our hybrid system in light of the requirement of high performance for information extraction tasks in the healthcare domain.

In this work, we present a hybrid method used for building the NLP component of an intelligent database system. In this first step, we are focusing only on FDA labels as the source of information. From the FDA labels, the system collects triples of information: [(1) drug name]-[(2) medical condition]-[(3) LOINC section header]. Table 1 provides examples of triples that are collected. This database will serve as one component of the hospital’s ADR detection pipeline. The hybrid method uses a supervised machine-learning algorithm – Conditional Random Fields (CRF) – coupled with handcrafted rules to collect the triples from the FDA drug labels’ narrative texts. The information includes data about medications and their corresponding medical conditions, such as indications, contraindications, and adverse reactions (manifested as disease/disorders and sign/symptoms). The reported work is a substantial step toward ADR detection by implementing an intelligent database.

**Table 1:** Excerpt of a drug label (Urea) annotated for three selected medical conditions and the conditions collected in three triples

			
**INDICATIONS AND USAGE**: For debridement and promotion of normal healing of hyperkeratotic surface lesions, particularly where healing is retarded by local infection, necrotic tissue, fibrinous or prurient debris or eschar. Urea is useful for the treatment of hyperkeratotic conditions such as dry, rough skin, dermatitis, psoriasis, xerosis, ichthyosis, eczema, keratosis, keratoderma, corns and calluses.	Drug*	**Condition**	LOINC Sections*
	Urea	hyperkeratotic lesion	INDICATION
	Urea	stinging	ADVERSE REACTION
**ADVERSE REACTIONS**: Transient stinging, burning, itching or irritation may occur and normally disappear on discontinuing the medication.	Urea	burning	ADVERSE REACTION
	**……**	**……**	**……**

*The drug names and LOINC Sections are added to the triples via rules that utilize the XML tags of the original label.

## Methods

### Data

#### Data set

The corpus is composed of 96,824 tokens or 52 FDA labels randomly selected from 37,907 FDA drug labels (accessed by 11/05/2012 on the DailyMed website) including 18,999 Rx drugs and 19,072 OTC drugs [21]. It covers three broad categories: OTC drugs (noted as OTC), Rx drugs from the top 200 drugs in sales from 2010 according to drugs.com [22] (noted as Rx_Top200), and other Rx drugs not part of the top 200 drugs in sales (noted as Rx_Other). Table 2 lists the names and corresponding National Drug Codes (NDCs) of five selected drug labels from each category. The research is exempt from human subjects research regulations. Consequently, IRB approval was not necessary.

**Table 2:** List of 15 selected drug labels

**Rx_Top200 (23)**		**Rx_Other (13)**	
**Drug Name**	**NDC**	**Drug Name**	**NDC**
Diovan	0083-4001-01	UREA	42192-101-10
ARICEPT	62856-851-30	GlucaGen HypoKit	0169-7065-15
DORYX	50546-550-01	Tramadol Hydrochloride	54868-4638-6
BENICAR HCT	65597-107-11	Lisinopril	51138-139-30
Copaxone	0088-1153-30	Glyburide	23155-058-10
**OTC (16)**			
**Drug Name**			**NDC**
Natural Fiber PowderOrange Flavor			53329-102-56
WhiskCare 373			65585-373-04
Degree for Men CleanAntiperspirant and Deodorant			64942-0866-2
UltrasolSunscreenSunscreen Lotion SPF 34			59886-319-11
Topcare Allergy			36800-479-68

The original drug labels are in XML format. We extracted the plain text version from the XML files. Although the FDA drug labels include 80 different Logical Observation Identifiers Names and Codes (LOINC) sections, we collected only the following eight medical-condition related sections as our source for medical-condition extraction, during the study: [23]

• Boxed Warning sections with the LOINC code of 34066–1

• Precautions sections with the LOINC code of 42232–9

• Warning and Precautions sections with the LOINC code of 43685–7

• Warning sections with the LOINC code of 34071–1

• Contraindications sections with the LOINC code of 34070–3

• Overdosage sections with the LOINC code of 34088–5

• Indications & Usage sections with the LOINC code of 34067–9

• Adverse reactions sections with the LOINC code of 34084–4

#### Gold standard annotation

Two categories of medical conditions were manually annotated: Disease/Disorders (DD) and Sign/Symptoms (SS). The guidelines for annotation were aligned with the Strategic Health IT Advanced Research Projects (SHARPn) Research Focus Area 4: Secondary Use of EHR Data’s annotations to provide future interoperability between our and the SHARPn clinical corpus [24]. All documents were double annotated by two native English speakers. One annotator had a clinical background (BSN, RN), and the other annotator had a Bachelor of Arts degree and extensive experience in annotating medical documents. The F-measure of inter-annotator agreement (IAA) is 0.85. Chapman et al. demonstrated that using both clinician and non-clinician annotators will not bias the annotated corpus, although non-clinicians need longer training times [25]. Disagreements were discussed and resolved by a third party; in our case, it was an NLP researcher. Details of the corpus and the process of gold standard development are thoroughly described in a separate paper [26]. The final gold standard was the result of consensus-seeking adjudication. Our corpus will be released publicly when the grant funded period ends (December 2013).

Table 1 shows an excerpt of an annotated drug label—Urea 40 Gel/Lotion. This drug label has two related LOINC sections – Indication and Adverse Reaction. The disease of *hyperkeratotic lesion* and sign/symptoms of *stinging* and *burning* are detected. With these extractions, the final triples are integrated, in this example as [Urea 40 Gel/Lotion]-[stinging]-[Adverse Reaction], and stored in the database according to the “[(1) drug name]-[(2) medical condition]-[(3) LOINC section header]” structure.

#### Descriptive statistics of the corpus

Table 3 shows the basic descriptive statistics of this corpus, including the number of annotated entities in the gold standard at token and span levels for each drug category. Because the original drug labels were in XML format, a simple parser was applied to extract the plain texts for these labels. The corpus of plain texts with eight related sections had 96,824 tokens (1,862 tokens per document). The OTC drugs have only two sections – Warning and Indications & Usages. These two sections are also the most frequent sections in the whole corpus. There are 8,611 annotated medical conditions in the corpus (121 for OTC; 2,091 for Rx_Top200; and 1,391 for Rx_Other); the average number of annotated medication conditions per document is 274.

**Table 3:** Descriptive statistics of medical conditions in the annotated drug labels

			**OTC**	**Rx_Top200**	**Rx_Other**	**ALL**
**Token**	**Disease/Disorder (DD)**	**All**	83	6,295	3,806	10,184
		**Unique**	46	1,129	737	1,423
	**Sign/Symptom (SS)**	**All**	104	2,072	1,867	4043
		**Unique**	46	525	437	742
	**Medical Conditions (DD&SS)**	**All**	187	8,367	5,673	14,227
		**Unique**	92	2,942	11,74	2,165
**Span**	**Disease/Disorder (DD)**	**All**	67	1,443	1271	2781
		**Unique**	39	553	470	860
	**Sign/Symptom (SS)**	**All**	54	3,642	2144	5840
		**Unique**	30	1,547	927	2114
	**Medical Conditions (DD&SS)**	**All**	121	5,085	3415	8,611
		**Unique**	69	2,091	1391	2,953

(Total=total number of tokens in that category, all=absolute number, unique = unique number).

Rx documents have more medical conditions than the OTC documents. The average number of medical conditions per document in Rx (128 for Rx_Top200 and 90 for Rx_Other) is 16 times greater than in OTC drugs (6 for OTC). Although the average of the DD entities (5 per document) is close to the average of the SS entities (7 per document) in OTC documents, the average of the DD entities (274 in Rx_Top200 and 293 in Rx_Other) is more than twice the average of SS entities (90 in Rx_Top200 and 144 in Rx_Other) in Rx documents. Approximately one-third of the medical conditions (8,611) are unique (2,953).

#### Evaluation method

For method development, the gold standard was divided into experimental and development sets. We held out 7,024 tokens and 74 annotated entities (or two documents randomly selected from the corpus) as the development set for manual error analysis. Although the number of named entities is large (8,611), they are distributed only in a smaller number of the documents (52), which serve as “containers” for the named entities. On average, the documents contained 166 annotated gold standard examples. However, some documents contained as many as 236 and some as few as 8 annotated gold standard examples. Splitting the documents into 10 subsets—required for 10-fold cross validation—would have created an uneven distribution of the annotated examples and, while some of the sets would have too many annotated gold standard examples, some sets would have included too few. To achieve more evenly distributed training and testing sets in the cross validation setting, we chose four-fold cross validation. That is, the gold standard set was randomly divided into four sets. Each subset included an average of 23,449 tokens and 2,134 annotated entities. Each time, the system trained on three subsets and tested on one subset. The testing subset is always different from the training sets to preserve the integrity of the testing, but each of the four subsets is rotated through as a test subset, while using the other three subsets for training. The reported results for the baseline and experimental systems are the averages of the four-fold cross validation sets. It is important to note that the sample size of the gold standard (and the performance of the machine learning algorithm) is based on the number of annotated tokens (8,611) and not the documents (52).

We measured standard NLP performance statistics, using precision/positive predictive value (*P*), recall/sensitivity (*R*), and F-measure (*F*), on both token and span levels [27,28]. *P* measures the number of true positive findings in relation to all findings, i.e., *P*=*TP*/(*TP*+*FP*) (where *TP* represents true positives and *FP* represents false positives). *R* measures the number of true positive findings in relation to all potential findings, i.e., R=*TP*/(*TP*+*FN*) (*FN* represents false negatives). F-measure is the harmonic mean of *R* and *P*, i.e., *F*=(2*P*R)/(*P*+*R*) [29]. The findings are presented for token level measurement (tokens in the documents) and span level measurement (spans or phrases in the documents). We did not use receiver operating characteristic (ROC) analysis, because it is not the most appropriate measure for the sequence-learning problem (such as named entity recognition in textual documents). ROC requires the number of negative cases to be computed, which is unknown in the case of named entities, as the entities are sequences of words [29]. Therefore, we used F-measure for the evaluation, as is standard.

Since the boundaries for medical conditions are often ambiguous, various matching criteria have been used on the span level. As such, we considered exact match, left match, right match, and partial match, as described by Tsai [30]. An exact match means the medical condition within the whole span is the same as the gold standard; the partial match criteria was also adopted because we assume that finding pieces of information is better than finding nothing at all. A left match is defined if the left boundary of the span matches exactly; a right match is defined if the right boundary of the span matches exactly; and a partial match is when any fragment in the span is correctly detected. Therefore, a left/right/partial match is helpful for indicating the performance of a system as well as for further error analyses. For example, if the system annotated only the token of “nausea” as sign/symptom for the sentence, “Side effects may include nausea and vomiting” (while the gold standard marked “nausea and vomiting” as a medical condition), then this extraction is correct on the left boundary detection but fails at the right boundary and is also considered a partial match.

We tested the statistical significance of the differences between the results of various system outputs using approximate randomization, which is not dependent on the underlying distribution of the data [31].

### A Hybrid pipeline

**Figure 1:**
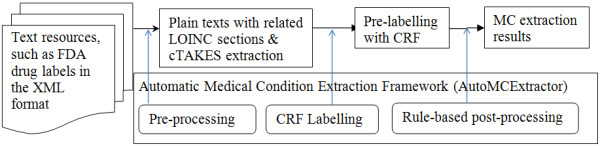
Automatic Medical Condition Extractor (AutoMCExtractor).

The hybrid pipeline for medical condition mining in FDA drug labels is called AutoMCExtractor. The first step is preprocessing; during this, plain texts with related LOINC sections are extracted from the XML files and cTAKES is executed on these text files to generate feature sets. The second step’s component uses a supervised learning method, CRF, to detect candidate medical conditions from the text excerpts. The final step’s component uses a dictionary-based approach to further improve the detection of medical conditions. Figure 1 depicts the pipeline in a graphical format.

#### Preprocessing

**Figure 2:**
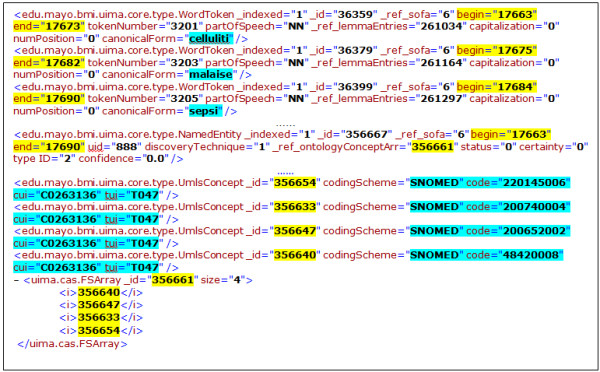
Excerpt of cTAKES Output.

The preprocessing step converts the input XML resources into plain texts and detects eight medical-condition-related LOINC sections in the XML files. We did not remove any stop words, nor did we do any stemming of the medical condition annotation and extraction; all of the medical conditions are annotated and extracted as they appeared in the documents. cTAKES is executed to generate the potential features for the medical condition identification. Simple linguistic features, such as tokens and POS, can be directly extracted from cTAKES results. The semantic features, such as SNOMED CD codes and TUIs (Type Unique Identifier), are also extracted from cTAKES results, but with an advanced algorithm. Figure 2 shows an example of a cTAKES output where the terms “cellulitis, malaise, sepsis” are linked to the SNOMED CD codes with the associated TUI code of “T047”. We used the following three steps to link “cellulitis” with the TUI code of T047 with cTAKES results: (1) cellulitis’s begin and end offsets are discovered by cTAKES as characters 17663 and 17673 and assigned to the ontology concept array ID 356661; (2) the ontology array’s concept ID (356661) is matched to the correct array that includes an array element that has a UIMA_UMLS ID of 356640; and (3) the 356640 UIMA_UMLS ID is then paired with an associated set of SNOMED CT, UMLS CUI and UMLS TUI codes.

#### CRF-based medical condition extraction

Selected components of the cTAKES output serve as partial input for the feature generation of the CRF supervised learning algorithm. We use MALLET’s CRF implementation [31]. Traditional BIO labels are used for the sequence labelling. That is, the beginning tokens of medical conditions are labelled as MC_B; the tokens within medical conditions are labelled as MC_I; and all other tokens are labelled as O. Different features are generated, including token features, linguistic features, and semantic features. Token features indicate the characteristics of the current token as well as its contexts, including the current token itself, the stem of the current token, the previous two tokens, and the next two tokens. Linguistic features include the Part-Of-Speech (POS) tags for the current token and POS tags for a 5-token window. Semantic features indicate whether the token belongs to certain thesauri, such as SNOMED CT. Table 4 lists the complete set of features used in the CRF algorithm, where the features in bold are utilized directly from the cTAKES output and the rest of the features are generated or modified by custom-developed processes.

**Table 4:** Feature sets for CRF

	
***Token features***	
Current token features	**The original form of the current token**, the lowercase form, and the stemmed form of the current token.
Tokens in the 5-window size	**The previous two tokens and the next two tokens in their original form**.
Bigram of current token	The current token bigram and the previous token bigram.
***Linguistic features***	
POS features	The **Part-Of-Speech (POS) of the tokens in a 5-token window**, including the current token, the previous two tokens, and the next two tokens.
Initial capital features	The features indicating whether the tokens (including the current token, the previous two tokens, and the next two tokens) are upper-case-initial.
Number or not features	The features indicating whether the current token is digital or alphabetic or mixed.
Capital feature	The feature indicating whether the current token is all capitalized or mixed with capital characters.
Prefix and suffix	The prefix and suffix of the current token (first or last two characters).
Token length	The character length of the current token.
***Semantic features***	
CUI	**The CUI code of the current token** from cTAKES by using dictionary based method.
TUI	**The TUI code of the current token** assigned by cTAKES, which provides the semantic type information contained in the UMLS thesaurus.

**Note: the features in bold** are utilized directly from the cTAKES output; the rest of the features are generated or modified by custom-developed processes.

#### Rule-based post-processing

Based on error analysis of the development set, we defined post-processing steps involving regular expressions to improve medical condition detection. It is a dictionary-based approach to complement the supervised learning algorithm. If the same medical condition occurs in the training set, then it will be annotated in the testing set, even though the CRF system does not recognize it. For example, “infection” and “photosensitivity” were considered to be medical conditions in the training set, although the CRF classifier may have failed to detect them; however, according to the post-processing rules, they were still labelled as medical condition during testing. If multiple medical conditions can be matched, then the maximum coverage rule will be applied. That is, the rule will only label the longest spans that match the medical conditions appearing in the training set. For example, for the phrase of “nausea and vomiting,” if “nausea,” “vomiting” and “nausea and vomiting” all exist in the corpus, then only “nausea and vomiting” are labelled as medical condition.

### Experimental settings

We built two rule-based baseline systems and three CRF-based experimental systems to evaluate our mining approach. The results of the baseline and experimental systems were compared in the evaluation phase against the gold standard test set. The five systems were trained and tested in a four-fold, cross-validation setting. Table 5 summarizes these five systems.

• **Baseline I**: a rule-based medical condition extraction method. This baseline assumes that semantic types assigned by cTAKES can identify the entities of DD and SS. The cTAKES TUIs lookup generated the UMLS TUI codes for the medical condition terms. These semantic types were selected based on the SHARPn guidelines to achieve consistency with the annotation from our gold standard [24]. The SHARPn guidelines excluded T033 (Finding) from the selection because it is a noisy semantic type and can correspond to several different classes such as Signs or Symptoms, Disease or Syndrome, or Lab Results.

• Since the TUIs and the entities of DD and SS are not in a one-to-one mapping, in the subsequent step, we used the following semantic types as medical conditions: Congenital Abnormality (with the TUI code of T019), Acquired Abnormality (with the TUI code of T020), Injury or Poisoning (with the TUI code of T037), Pathologic Function (with the TUI code of T046), Disease or Syndrome (with the TUI code of T047), Mental or Behavioral Dysfunction (with the TUI code of T048), Cell or Molecular Dysfunction (with the TUI code of T049), Experimental Model of Disease (with the TUI code of T50), Signs and Symptoms (with the TUI code of T184), Anatomical Abnormality (with the TUI code of T190), and Neoplastic Process (with the TUI code of T191).

• **Baseline II**: another rule-based extraction system, where all terms in the test set were tagged as medical conditions, if the same term also appeared in the training set. The pattern matching approach was performed according to the longest exact match method. It assumed the system had a dictionary (generated from the training set) and used this dictionary to detect medical conditions in the testing set. This is considered to be a pattern matching approach.

• **Experimental I:** an implementation of the AutoMCExtractor system. The system uses MALLET CRF for the supervised sequence learning with the basic feature set of token features and linguistic features, as described in Table 4. These features were used in our previous project for medication name detection in clinical notes and showed excellent performance.

• **Experimental II**: an implementation of our AutoMCExtractor system. The system uses MALLET CRF for the supervised sequence learning with the same token and linguistic features as in Experimental I but added TUIs as features, as described in Table 4.

• **Experimental III**: an implementation of our AutoMCExtractor system. The system uses MALLET CRF with token and linguistic features but added CUIs, as described in Table 4. TUIs are not included as a feature in this experiment.

**Table 5:** Features in baseline and experimental systems

	**Dictionary**	**Token features**	**Linguistic features**	**Semantic features**	
				**TUI**	**CUI**
**Baseline I**				X	
**Baseline II**	X				
**Experiment I**	X	X	X		
**Experiment II**	X	X	X	X	
**Experiment III**	X	X	X		X

## Results

Evaluation results on both token and span levels are shown in Table 6. Since the two baseline systems use rule-based methods, only exact match results are reported on the span level. For baseline systems, Baseline II performs better (0.888, 0.698, and 0.781 for P, R, and F-measure respectively) than Baseline I (0.827, 0.506, and 0.628) on the token level evaluation.

**Table 6:** The token and span level results of the experiment

			**Precision**	**Recall**	**F-measure**
**Baseline I**	Token	MC_B	0.661	0.575	0.615
		MC_I	0.890	0.21	0.338
		Overall	0.775	0.391	0.476
	Span	Exact Match	0.827	0.506	0.628
**Baseline II**	Token	MC_B	0.804	0.733	0.767
		MC_I	0.811	0.473	0.597
		Overall	0.808	0.603	0.681
	Span	Exact Match	0.888	0.698	0.781
**Experiment I**	Token	MC_B	0.910	0.782	0.841
		MC_I	0.936	0.660	0.773
		Overall	0.919	0.731	0.814
	Span	Exact Match	0.886	0.766	0.822
		Left Match	0.915	0.862	0.888
		Right Match	0.941	0.877	0.908
		Partial Match	0.982	0.849	0.911
**Experiment II**	Token	MC_B	0.928	0.831	0.877
		MC_I	0.942	0.686	0.793
		Overall	0.933	0.771	0.844
	Span	Exact Match	0.900	0.812	0.854
		Left Match	0.931	0.841	0.886
		Right Match	0.944	0.852	0.900
		Partial Match	0.985	0.889	0.935
**Experiment III**	Token	MC_B	0.912	0.787	0.845
		MC_I	0.936	0.663	0.775
		Overall	0.920	0.735	0.817
	Span	Exact Match	0.886	0.769	0.824
		Left Match	0.917	0.844	0.879
		Right Match	0.944	0.861	0.900
		Partial Match	0.982	0.852	0.912

For experimental systems, Experiment II has the highest performance on both the token (0.933, 0.771, and 0.844) and span (0.90, 0.812, and 0.854) levels (exact match). Experiment III shows the second best performance (0.921, 0.734, and 0.817 on the token level; and 0.886, 0.769, and 0.824 for the span level, exact match). Experiment I has P, R, and F-measure of 0.919, 0.731, and 0.814, respectively, on the token level; and 0.886, 0.766, and 0.822, respectively, at the exact span match level. The three experimental systems perform statistically significantly better than the two baseline systems (p<0.05) on the span level as tested by approximate randomization, as shown in Table 7.

**Table 7:** The statistical significance tests (with p-values < 0.007)

	**Precision**	**Recall**	**F-measure**
**Baseline I vs. Baseline II**	**<0.0001**	**<0.0001**	**<0.0001**
**Baseline II vs. Experiment I**	**0.003**	**<0.0001**	**<0.0001**
**Baseline II vs. Experiment II**	**0.001**	**<0.0001**	**<0.0001**
**Baseline II vs. Experiment III**	**0.0018**	**<0.0001**	**<0.0001**
**Experiment I vs. Experiment II**	0.039	**<0.0001**	**<0.0001**
**Experiment I vs. Experiment III**	0.147	0.0409	0.0264
**Experiment II vs. Experiment III**	0.215	**<0.0001**	**<0.0001**

Note 1: Statistical significance was tested using approximate randomization [32].

Note 2: Bonferroni correction was applied because of multiple comparisons [33].

In the best-performing system, Experiment II, the partial match performance scores (0.985, 0.889, and 0.935) are higher than the right match (0.944, 0.852, and 0.903) scores and left match (0.931, 0.840, and 0.891). All of these matches have higher scores than the exact match. The same trend also exists in the other experimental systems, Experiment I and Experiment III.

The corresponding p-values for the span-level, statistical-significance tests are shown in Table 7. Due to the number of different tests conducted, we applied a Bonferroni correction to account for the increased possibility of Type I error. Thus, to adjust for 7 different significance tests with multiple variables that may not be independent, the performance was considered statistically significant at p-values lower than 0.007, i.e., 0.05/7 [33]. Baseline I versus Baseline II, Baseline II versus Experiment I, Baseline II versus Experiment II, Baseline II versus Experiment III, and Experiment I versus Experiment II have p-value lower than 0.007 among the precision statistical significance tests. All the recall and F-measure comparisons are found to be statistically significant, except in the comparison of Experiment I and Experiment III.

## Discussion

We reported the average of four-fold cross validation performance statistics. As shown in Tables 6 and 7, Baseline II is statistically significantly better than Baseline I in precision, recall and F-measure, according to the approximate randomization test. Therefore, we can conclude that the dictionary-based pattern matching approach (Baseline II), i.e., tagging all terms by longest exact match in the test set if the matching terms were also tagged in the training set, is better than the TUI-based extraction approach (Baseline I).

The experimental systems are statistically significantly better than the baseline systems. That means that our system, based on the CRF method with both linguistic and dictionary features, achieves better performance than either the solely dictionary-based or TUI-based extraction systems. The system of Experiment II is statistically significantly better than the Experiment I system (for recall and F-measure), which indicates that the TUI feature helps in identifying more medical conditions with higher accuracy. Although Experiment III has statistically significantly better performance than Experiment II on recall and F-measure, it is not significantly improved with respect to precision. This means that the CUI feature is not useful in finding more medical concepts.

The error analysis on the development set showed that the false positives were mainly due to boundary-detection errors (five out of seven). For instance, in the phrase “generalized rash”, both “generalized” and “rash” were tagged by the system, while only “rash” was tagged in the gold standard as a medical condition (“generalized” is considered an attribute in the gold standard corpus). False negatives were mostly single-word concepts that were missed by the system (three out of four). For example, “insulinoma” was not detected by our system.

In the three experimental systems, the performance of partial match is better than right match, right match is better than left match, and left match is better than exact match in P, R, and F (i.e., partial match > right match > left match > exact match). Exact match is a stricter measurement than partial match—it requires that both the left boundary and the right boundary be correct. For example, if the gold standard annotates “generalized rash” as medical condition (while the system only identifies “rash”), then it is a partial match and right match, but neither a left match nor exact match. This can diminish both precision and recall for the left match and exact match evaluation. The F-measure for the partial match is greater than 0.91, which demonstrates that the system can detect the medical conditions very well, at least partially. However, the F-measure for exact match is only 0.82, which means that the system fails at boundary detections (especially the left boundary detection), since the performance of right match is better than left match. In error analysis, non-matches for the left boundary were seen with modifiers in front of the medical concepts, such as “inflammatory lesions.”

Precision of token-level evaluation is better than the span-level exact match, but the span-level exact match is better than token-level exact match for recall and F-measure for all three experimental systems. Therefore, in this hybrid system, recall has a greater effect on overall system performance (F-measure) than precision.

One of the limitations of our current study is that we did not take into account dis-continuous entities, such as “muscle…weakness” in “muscle tenderness or weakness”. Discontinuous entities are hard to represent accurately in the input format of a standard CRF sequence labelling system, i.e., BIO format. They represent about five percent of all annotated entities. This limitation also explains the performance difference between detecting beginning tokens of medication conditions (i.e., MC_B) and left match. In the continuous and non-overlapping named entity detection, these two scores should be exactly the same. However, in an overlapping case, we count only once for the token level, but multiple times for the span-level left match. For example, both “muscle tenderness” and “muscle…weakness” in “muscle tenderness or weakness” are annotated in the gold standard. For the token-level evaluation, MC_B is counted as one (i.e., “muscle”), while span-level left match is counted as two, since there are two medical conditions.

The current system performance is good (the overall F-measure is 0.89 for exact match). This is especially true compared with the inter-annotator agreement (F-measure of 0.85). In essence, the system recognizes the medical conditions as well as the human annotators and behaves as a third human annotator. However, the recall is still relatively low (0.889 for partial match of Experiment III). If we exclude the discontinuous named entities (about 5%), the recall is around 0.94. In our future work, we will further investigate methods for improving the coverage for medical conditions. Moreover, we will address discontinuous medical condition detection, which will most likely be identified through post-processing rules or an ensemble machine learning approach.

In the next phase of development, we will test the full pipeline on a selected set of drugs to directly monitor ADRs, which we collect from FDA labels in the EHR. However, that system will require development of further components of the ADR monitoring pipeline (including a component that extracts relevant data from the EHR). We anticipate that especially in the beginning, the intelligent database system of which the NLP system described here will be a component will need human supervision, but we are also planning to develop additional components for the data collection from diverse information sources (e.g. journal texts/abstracts and the AERS data set) and use statistical cross-referencing to increase the reliability of the collected and stored “drug/medical condition/LOINC header” triples.

## Conclusions

This study offers three main contributions to the literature. First, we demonstrated that medical conditions could be extracted from FDA drug labels, which is a ubiquitous source of information, with high performance using scalable NLP methods. Second, incorporating the open source cTAKES clinical NLP system into our pipeline, we explored the features used for detecting medical conditions in sequence learning. To our knowledge, this is the first study that makes comprehensive use of linguistic and semantic processing from cTAKES outside of the domain of clinical notes and in combination with *de novo* generated features that are incorporated into feature vectors for a machine learning algorithm. Although many sequence learning tasks, especially named entity recognition, use CRF algorithms, the feature details are rarely discussed in the literature and complete evaluations are not available for clinical notes. Third, the AutoMCExtractor serves as a proof of the concept that an intelligent database of drugs and corresponding medical conditions can be built. Although collecting “drug/medical condition/LOINC” triples is only the first step in developing an intelligent ADR detection database, it is nonetheless an important step toward large-scale automated ADR detection.

## Abbreviations

CCHMC: Cincinnati children’s hospital medical center (CCHMC); FDA: Food and drug administration; ADR: Adverse drug reaction; Rx: Prescription drugs; OTC: Over-the-counter drugs; MedDRA: Medical dictionary for regulatory activities; AR: Adverse reactions; AERS: Adverse event reporting system; CRF: Conditional random field; NLP: Natural language processing; LOINC: Logical observation identifiers names and codes; cTAKES: clinical text analysis and knowledge extracting system (cTAKES); NDCs: National drug codes; DD: Disease/disorders; SS: Sign/symptoms; POS: Part-of-speech; CUI: Concept unique identifier; TUI: semantic type unique identifier; P: Precision; R: Recall; F: F-measure; TP: True positive; FP: False positive; FN: False negative; ROC: Receiver operating characteristic.

## Competing interests

The authors declare that they have no competing interests.

## Authors’ contributions

QL designed the study: downloaded the FDA drug labels; cleaned the corpus; pre-processed the documents using cTAKES; built the pipeline system, autoMCExtractor; conducted the experiments; performed the manual data evaluation; and wrote the original manuscript. LD provided guidance in the gold standard vetting process and supervised the development of the gold standard. MK and LS conducted the whole annotation task. HZ and TL contributed ideas and helped in system implementation, and HZ, TL, and KBC contributed ideas for algorithm development. This project was supervised by IS. This manuscript was prepared by QL and IS with additional contributions by all authors. All authors read and approved the final manuscript.

## Pre-publication history

The pre-publication history for this paper can be accessed here:

http://www.biomedcentral.com/1472-6947/13/53/prepub

## References

[B1] Why learn about Adverse Drug Reactions (ADR)? http://www.fda.gov/Drugs/DevelopmentApprovalProcess/DevelopmentResources/DrugInteractionsLabeling/ucm114848.htm

[B2] Le J Nguyen T Law AV Hodding J Adverse drug reactions among children over a 10-year period Pediatrics 2006 118 2 555 562 10.1542/peds.2005-2429 16882807

[B3] Muething SE Conway PH Kloppenborg E Lesko A Schoettker PJ Seid M Kotagal U Identifying causes of adverse events detected by an automated trigger tool through in-depth analysis Qual Saf Health Care 2010 19 5 435 439 10.1136/qshc.2008.031393 20798069

[B4] Adverse Event Reporting System (AERS) http://www.fda.gov/Drugs/GuidanceComplianceRegulatoryInformation/Surveillance/AdverseDrugEffects/

[B5] FDA Label http://www.fda.gov/ScienceResearch/BioinformaticsTools/ucm289739.htm

[B6] Boyce R Harkema H Conway M **Leveraging the semantic web and natural language processing to enhance drug-mechanism knowledge in drug product labels** Proceedings of the First ACM International Health Informatics Symposium: November 11 - 12, 2010 2010 Arlington, VA, USA 492 496

[B7] Chen M Vijay V Shi Q Liu Z Fang H Tong W FDA-approved drug labelling for the study of drug-induced liver injury Drug Discov Today 2011 16 697 703 10.1016/j.drudis.2011.05.007 21624500

[B8] Murphy S Roberts R “Black box” 101: How the Food and Drug Administration evaluates, communicates, and manages drug benefit/risk J Allergy Clin Immunol 2006 117 1 34 39 10.1016/j.jaci.2005.10.031 16387581

[B9] Jha AK Kuperman GJ Teich JM Leape L Shea B Rittenberg E Burdick E Seger DL Vander Vliet M Bates DW Identifying adverse drug events: development of a computer-based monitor and comparison with chart review and stimulated voluntary report J Am Med Inform Assoc 1998 5 3 305 314 10.1136/jamia.1998.0050305 9609500 PMC61304

[B10] Phansalkar S Hoffman JM Nebeker JR Hurdle JF Pharmacists versus nonpharmacists in adverse drug event detection: a meta-analysis and systematic review Am J Health Syst Pharm 2007 64 8 842 849 10.2146/ajhp060335 17420201

[B11] Coding Symbols for Thesaurus of Adverse Reaction Terms (COSTART) Source Information. (2010, November 23) U.S National Library of Medicine http://www.nlm.nih.gov/research/umls/sourcereleasedocs/current/CST/

[B12] Bousquet C Lagier G Louet AL Beller C Venot A Jaulent M Appraisal of the MedDRA Conceptual Structure for Describing and Grouping Adverse Drug Reactions Drug Saf 2005 28 1 19 34 10.2165/00002018-200528010-00002 15649103

[B13] He Y Xiang Z Sarnitivijai S Toldo L Ceusters W **AEO: a realism-based biomedical ontology for the representation of adverse events** Proceedings of International Conference on Biomedical Ontology (ICBO): July 24-26, 2009 2009 Amherst, New York, USA 309 315

[B14] Alecu I Bousquet C Jaulent MC A case report: using SNOMED CT for grouping adverse drug reactions terms BMC Med Inform Decis Mak 2008 8 54 10.1186/1472-6947-8-54 19007441 PMC2582791

[B15] Sohn S Kocher JPA Chute CG Savova GK Drug side effect extraction from clinical narratives of psychiatry and psychology patients J Am Med Inform Assoc 2011 18 Suppl 1 144 149 10.1136/amiajnl-2011-000351 PMC3241172 21946242

[B16] Duke JD Friedlin J **ADESSA: a real-time decision support service for delivery of semantically coded adverse drug event data** Proceedings of AMIA Annual Symposium 2010 Washington, DC, USA 177 181 PMC3041415 21346964

[B17] Savova GK Masanz JJ Ogren PV Zheng J Shon S Kipper-Schuler KC Chute CG Mayo clinical Text Analysis and Knowledge Extraction System (cTAKES): Architecture, component evaluation and applications J Am Med Inform Assoc 2010 17 5 507 513 10.1136/jamia.2009.001560 20819853 PMC2995668

[B18] Bisgin H Liu Z Fang H Xu X Tong W Mining FDA drug labels using an unsupervised learning technique - topic modeling BMC Bioinforma 2011 12 Suppl 10 S11 10.1186/1471-2105-12-S10-S11 PMC3236833 22166012

[B19] Li Q He D Mao M **A study of relation annotation in business environments using web mining** Proceeding of International Conference on Semantic Computing 2009 Berkeley, CA, USA 203 209

[B20] Aramaki E Miura Y Tonoike M Ohkuma T Masuichi H Wakiki K Ohe K Extraction of adverse drug effects from clinical records Stud Health Technol Inform 2010 160 1 739 743 20841784

[B21] DailyMedhttp://dailymed.nlm.nih.gov/dailymed/about.cfm

[B22] Pharmaceutical Sales 2010. - Top U.S. Drugs by Saleshttp://www.drugs.com/top200.htm

[B23] Structured Product Labeling, Section Headings (LOINC) http://www.fda.gov/ForIndustry/DataStandards/StructuredProductLabeling/ucm162057.htm

[B24] SHARPn annotation guidelines[www.sharpn.org]. (Oral communication from SHARPn’s NLP PI)

[B25] Chapman WW Dowling JN Hripcsak G Evaluation of training with an annotation schema for manual annotation of clinical conditions from emergency department reports Int J Med Inform 2008 77 2 107 113 10.1016/j.ijmedinf.2007.01.002 17317291

[B26] Deleger L Li Q Lingren T Kaiser M Molnar K Zhai H Stoutenborough L Solti I **Building Gold standard Corpora for Medical Natural Language Processing Tasks** Proceedings of AMIA Annual Symposium 2012 Chicago, USA 144 153 PMC3540456 23304283

[B27] Uzuner O Solti I Cadag E Extracting medication information from clinical text J Am Med Inform Assoc 2010 17 5 514 518 10.1136/jamia.2010.003947 20819854 PMC2995677

[B28] Friedman C Hripcsak G Evaluating natural language processors in the clinical domain Methods Inf Med 1998 37 4–5 334 344 9865031

[B29] Hripcsak G Rothschild AS Agreement, the f-measure, and reliability in information retrieval J Am Med Inform Assoc 2005 12 3 296 308 10.1197/jamia.M1733 15684123 PMC1090460

[B30] Tsai R Wu S Chou W Lin Y He D Hsiang J Sung T Hsu W Various criteria in the evaluation of biomedical named entity recognition BMI Bioinformatics 2006 7 92 100 10.1186/1471-2105-7-92 PMC1402329 16504116

[B31] Noreen EW Computer-Intensive Methods for Testing Hypotheses: an Introduction 1989 New York: Wiley

[B32] MALLET: A MAchine Learning for Language Toolkithttp://mallet.cs.umass.edu/

[B33] Bland JM Altman DG Multiple significance tests: the Bonferroni method BMJ 1995 310 6973 170 10.1136/bmj.310.6973.170 7833759 PMC2548561

